# Interspecies cathelicidin comparison reveals divergence in antimicrobial activity, TLR modulation, chemokine induction and regulation of phagocytosis

**DOI:** 10.1038/srep40874

**Published:** 2017-01-19

**Authors:** Maarten Coorens, Maaike R. Scheenstra, Edwin J. A. Veldhuizen, Henk P. Haagsman

**Affiliations:** 1 Department of Infectious Diseases and Immunology, Division of Molecular Host Defence, Faculty of Veterinary Medicine, Utrecht University, Yalelaan 1, 3584 CL Utrecht, The Netherlands

## Abstract

Cathelicidins are short cationic peptides initially described as antimicrobial peptides, which can also modulate the immune system. Because most findings have been described in the context of human LL-37 or murine CRAMP, or have been investigated under varying conditions, it is unclear which functions are cathelicidin specific and which functions are general cathelicidin properties. This study compares 12 cathelicidins from 6 species under standardized conditions to better understand the conservation of cathelicidin functions. Most tested cathelicidins had strong antimicrobial activity against *E. coli* and/or MRSA. Interestingly, while more physiological culture conditions limit the antimicrobial activity of almost all cathelicidins against *E. coli*, activity against MRSA is enhanced. Seven out of 12 cathelicidins were able to neutralize LPS and another 7 cathelicidins were able to neutralize LTA; however, there was no correlation found with LPS neutralization. In contrast, only 4 cathelicidins enhanced DNA-induced TLR9 activation. In conclusion, these results provide new insight in the functional differences of cathelicidins both within and between species. In addition, these results underline the importance not to generalize cathelicidin functions and indicates that caution should be taken in extrapolating results from LL-37- or CRAMP-related studies to other animal settings.

Cathelicidins are cationic peptides with an important function in the early vertebrate host response against invading pathogens^1^. They are secreted at mucosal surfaces and, during infection, by leukocytes and epithelial cells upon interaction with microbes. Cathelicidins have both direct antimicrobial activity as well as immunomodulatory functions^2^,^3^,^4^,^5^,^6^,^7^. The importance of cathelicidins in innate host defense has been demonstrated in mice lacking cathelicidin expression. These mice have an increased susceptibility for various pathogens^8^,^9^,^10^,^11^. In addition, cathelicidins have been shown to have therapeutic potential. Overexpression of cathelicidin in a lung xenograft model has been shown to promote *P. aeruginosa* and *S. aureus* killing^12^, while exogenous cathelicidin treatment has been successfully used to inhibit *M. haemolytica, E. coli* and *S. aureus* infections^13^,^14^,^15^.

Cathelicidins are found in most vertebrate species and contain a highly conserved cathelin-domain, which is cleaved off during secretion, releasing the active peptide. Although these active peptide sequences are highly variable between species, many cathelicidins have the ability to adopt an amphipathic α-helical structure^16^. Most cathelicidins have been described in the context of their antimicrobial activity, while various other functions have been identified for a limited number of cathelicidins^17^ including induction of chemokine expression^18^, intrinsic chemotactic activity^19^, neutralization of LPS-induced TLR4 activation and LTA-induced TLR2 activation^18^,^20^,^21^, enhancement of DNA-induced plasmacytoid DC and macrophage activation^22^,^23^, promotion of wound healing^24^, influencing DC and macrophage differentiation^25^,^26^ and regulation of phagocytosis^27^. However, even the most well-described functions are often tested under different conditions, making it difficult to compare properties between cathelicidins. In addition, because several functions have only been described for a limited number of cathelicidins, it is unclear which properties are peptide-specific and which are related to general functions of cathelicidins.

In this study, 12 cathelicidins from 6 different species were selected to assess their ability to exert various well-known cathelicidin functions. Our results show that various functions, including antimicrobial activity and LPS neutralization, are prevalent for most, although not for all, cathelicidins. In contrast, direct chemokine induction and enhancement of DNA activation of RAW264.7 cells were only observed for a few cathelicidins and only at relatively high concentrations. In total, these results provide novel insights in the functional differences between cathelicidins and could prove useful in the development of new cathelicidin-based anti-infective therapies.

## Materials and Methods

### Reagents

TLR ligands: LPS *Escherichia coli (E. coli*) O111:B4 (TLR-4), LTA *S. aureus* (TLR-2), and ODN-1826 (TLR-9) were obtained from Invivogen (Toulouse, France). Chicken CATH-2 (chCATH-2) and PMAP-36 were synthesized by Fmoc-chemistry at China Peptides (CPC scientific, Sunnycale, CA) and all other cathelicidins were synthesized by Fmoc-chemistry at the Academic Centre for Dentistry Amsterdam (Amsterdam, The Netherlands) (Table 1). Purity of all peptides was >95% as analyzed by HPLC-MS.

### Cell and bacterial culture

*E. coli* O78 (Zoetis Animal Health, Kalamazoo, MI, USA), *E. coli ATCC 25922, Staphylococcus aureus ATCC 29213*, and methicillin resistant *Staphylococcus aureus* (MRSA) (WKZ-2, human clinical isolate) were grown overnight from a glycerol stock in Mueller Hinton Broth (MHB) (Becton Dickinson, USA). Before use, bacteria were grown to mid-log phase in MHB for 2 hours at 37 °C, 200 RPM. Murine RAW264.7 macrophages (ATCC-TIB-71) were obtained from the American Type Culture Collection (ATCC, Manassas, VA, USA) and cultured in DMEM (41966-029; Thermo Fisher Scientific, Waltham, MA, USA) supplemented with 10% FCS (Bodinco B.V., Alkmaar, the Netherlands) (DMEM + FCS) at 37 °C, 5.0% CO_2_. Cells were seeded in 96-wells plates at 5 × 10^5^ cells/ml or 12-wells plates at 2 × 10^5^/ml for adherence overnight prior to stimulation.

### Antimicrobial activity

Peptides (0.63 to 40 μM) and bacteria mid-log cultures (2 * 10^6^ CFU/ml) were prepared in MHB or DMEM + FCS and mixed (100 μl peptide + 100 μl bacterial culture) in a Bioscreen C analyzer plate (Oy Growth Curves Ab Ltd, Helsingfors, Finland). Plates were placed in a Bioscreen C analyzer (Oy Growth Curves Ab Ltd) and bacteria were grown for 16 h at 37 °C with 200 RPM. The OD was measured every 15 minutes using a wideband filter (450–580 nm) to measure bacterial growth. The time needed grow above an OD of 0.6 was determined for all concentrations. Activity of peptides was expressed as the C_max_, the concentration of peptide required to delay the growth for 10 h compared to the control. Or, for peptides with low activity (no C_max_), activity was expressed as the hours of growth delay at 20 μM. Unlike the classic MIC and MBC determination used in microbiology, this method also enabled comparison of peptides with low antibacterial activity.

In order to compare our C_max_ with MBC values, wells without visible bacterial growth were plated out on TSA plates and incubated o/n at 37 °C to determine the presence of viable bacteria.

### Sytox green assay

Peptide dilutions and bacteria suspensions were prepared as described above for testing the antimicrobial activity in the Bioscreen C analyzer. Peptide dilutions and bacteria were mixed 1:1 and incubated for 30 minutes at 37 °C. Next, bacteria were washed once with PBS and transferred to black 96-wells assay plates (Corning, OH, USA). Bacteria were incubated with 1 mM sytox green (Life technologies) (λ_ex_ 504 nm and λ_em_ 523 nm) for 5 minutes after which fluorescence was measured using a FLUOstar Omega microplate reader (BMG Labtech GmbH, Ortenberg, Germany).

### Cytotoxicity

WST-1 reagent was obtained from Roche (Basel, Switzerland). RAW264.7 cells were incubated with cathelicidins for 24 h, after which the supernatant was removed and replaced with 10% WST-1 reagent in culture medium. After 20 minutes, absorbance was measured at 450 nm with a FLUOstar Omega microplate reader (BMG Labtech GmbH, Ortenberg, Germany) and was corrected for absorbance at 630 nm. Non-treated control cells were defined as 100% mitochondrial activity.

In addition, cells were detached after peptide exposure and stained with propidium iodide (PI) (BD bioscience, San Jose, CA, USA). Percentages of PI positive (*i*.*e*. dead) cells were determined with flow cytometry (BD FACSCanto II flow cytometer (BD Biosciences)) and analyzed with FlowJo software (Ashland, OR, USA).

### TLR stimulation

RAW264.7 cells were stimulated with 100 ng/ml LPS *E. coli* O111:B4, 1 μg/ml LTA *S. aureus* or 2.5 nM ODN-1826 in the presence of various concentrations of different cathelicidins. TNFα release was determined after 2 h for LPS and LTA stimulation and 24 h for ODN-1826 stimulation. CXCL10, CCL5 and IL-10 release were all determined after 24 h stimulation. As a control, RAW264.7 cells were stimulated for 2 h with 10^6^ CFU/ml live or heat-killed (70 °C, 0.5 h) *E. coli* O78, followed by a double wash with cell culture medium and subsequent 22 h incubation in cell culture medium supplemented with 250 μg/ml gentamicin.

### ELISA

ELISA Duoset kits for mouse TNFα, CCL5, CXCL10 and IL-10 were obtained from R&D systems (Minneapolis, MN, USA) and ELISAs were performed according to the manufacturer’s protocol. Samples were stored at −20 °C until analysis and, if needed, diluted in 1% BSA in PBS, pH 7.4. Absorbance at 450 nm was determined in a FLUOstar Omega microplate reader (BMG Labtech GmbH) and corrected for absorbance at 570 nm. Results were analyzed with MARS data analysis software (BMG Labtech GmbH).

### Phagocytosis assay

Red fluorescent (λ_ex_ 575 nm and λ_em_ 610 nm) carboxylate-modified polystyrene latex beads (0.5 μm; Sigma Aldrich, St. Louis, MO, USA) were washed three times with PBS and resuspended in culture medium. Peptide dilutions were prepared in culture medium and added to RAW264.7 cells, directly followed by the latex beads (ratio 10 beads to 1 cell). Cells were incubated for 30 minutes at 37 °C, 5% CO_2_ (energy-dependent uptake) or 0 °C (non-specific adherence), after which cells were washed extensively with ice-cold PBS supplemented with 1% FCS and 0.01% NaN_3_, to remove all free beads. After washing, cells were scraped and resuspended in FACS buffer (PBS supplemented with 0.5% BSA). Samples were measured with the BD FACSCanto II flow cytometer (BD Biosciences, San Jose, CA, USA) and analyzed with FlowJo software (Ashland, OR, USA). Mean fluorescence intensity (MFI), corrected for non-specific adherence, was used as an indicator for the number of beads taken up.

### Statistics

Results are presented as the mean ± standard error of the mean (SEM) of at least three independent experiments. Statistical significance was assessed with Two-way ANOVA followed by the Bonferroni Post-Hoc test in Prism software, version 6.02 (GraphPad Prism, La Jolla, CA, USA). All samples were compared to 0 μM controls. *p < 0.05; **p < 0.01; ***p < 0.001.

## Results

### Antibacterial activity of cathelicidins

**Figure 1:**
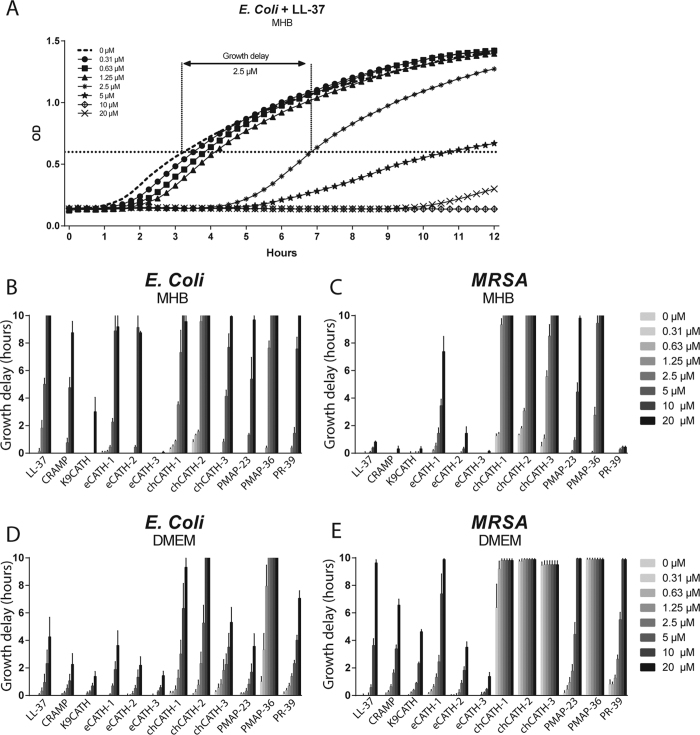
Antibacterial activity of cathelicidins. *E. col*i and MRSA (1 × 10^6^ CFU/ml) of were grown in MHB or DMEM ± FCS for 16 hours under constant shaking (200 RPM). Every 15 minutes the OD was measured. Growth delay was defined as the time needed for peptide-treated bacteria to grow above an OD of 0.6 compared to the control bacteria (no peptide added) (**A**). Cathelicidins were tested in different concentrations (0.31 μM, 0.63 μM, 1.25 μM, 2.5 μM, 5 μM, 10 μM, and 20 μM) to determine the antimicrobial activity in MHB against *E. coli* (**B**) or MRSA (**C**) and in more physiological medium DMEM + FCS (**D**,**E**). Results are presented as average +/− SEM (N = 4).

Twelve cathelicidins from 6 different species were selected for this study, namely: human LL-37, murine CRAMP, dog K9CATH, equine CATH (eCATH)-1, -2 and -3, chicken CATH (chCATH)-1, -2, and -3, porcine PMAP-23 and -36 and PR-39 (Table 1). Antimicrobial activity against *E. coli* and MRSA was directly compared under standardized conditions in MHB, a medium optimized for bacterial growth. Bacterial growth delay was defined as the delay in hours for peptide-treated bacteria to reach an OD of 0.6, compared to the non-treated control. C_max_ is the minimal concentration needed to delay growth for a minimum of 10 hours compared to the non-treated control (Table 1 and Fig. 1A).

Ten out of 12 cathelicidins strongly delayed the growth of *E. coli*, with a growth delay of 9 h or more, with chCATH-2 and PMAP-36 as most potent, with a C_max_ of 5 μM. Only K9CATH and eCATH-3 showed no or very little activity (Fig. 1B). MRSA was more resistant to most peptides than *E. coli*. Only chCATH-1, -2, and -3 were more active against MRSA than against *E. coli*. In addition to the three chicken cathelicidins, PMAP-23 and -36 had a C_max_ of 20 μM and 10 μM respectively and eCATH-1 delayed MRSA growth with 7.5 hours at 20 μM. However, none of the other peptides were active against MRSA in MHB (Fig. 1C).

While these testing conditions are widely used to determine antimicrobial activity, they poorly represent physiological conditions. Since it has been shown that serum components and salts can have an inhibitory effect on antimicrobial activity^28^,^29^,^30^, growth inhibition of *E. coli* and MRSA was also assessed in DMEM + FCS, which better represents physiological conditions. Under these conditions only chCATH-1, and -2, PMAP-36 and PR-39 were able to delay the growth of *E. coli* at least 7 hours. Interestingly, PMAP-36 was the only peptide with an increased activity against *E. coli* in DMEM + FCS compared to MHB (Fig. 1D). In contrast to *E. coli*, antimicrobial activity against MRSA was enhanced for all 12 cathelicidins in DMEM + FCS. This was most pronounced for chCATH-2 and -3 and PMAP-36, with a C_max_ of 0.31 μM, which is the lowest concentration tested (Fig. 1E). In addition, while PR-39 had no effect on MRSA in MHB, it was more potent in DMEM + FCS with a C_max_ of 10 μM. In order to rule out bacterial strain specific effects, peptides were also tested against *E. coli* ATCC 25922 and *S. aureus* ATCC 29213. Very similar results, with a maximal 2 fold difference in C_max_ were obtained for all peptides, except for PR-39 which showed an 8–16 fold decrease in C_max_ value. Overall though, very little bacterial strain variability was observed (Supplementary Fig. S1).

The capacity of peptides to induce membrane leakage was tested using the permeability marker sytox green. For *E. coli*, all peptides that caused growth delay also caused membrane permeability, with the exception of PR-39 which showed only a minimal permeability at its C_max_ concentration (Supplementary Fig. S2). For MRSA, fluorescence levels were generally very low even at >C_max_ levels, hampering a good comparison between growth delay and permeabilization for this strain.

### Cathelicidin induced chemokine and cytokine release by RAW264.7 cells

To determine the direct effect of cathelicidins on chemokine induction, RAW264.7 cells were used, which have previously been shown to increase chemokine secretion upon stimulation with LL-37^18^. Since cathelicidins have membrane-perturbing properties which might affect the host’s cell membrane^15^,^31^,^32^,^33^,^34^,^35^, first the possible cytotoxicity of the cathelicidins to RAW264.7 cells was assessed. No or very limited cytotoxicity was observed for most cathelicidins. chCATH-1 and PMAP-36 showed some cytotoxicity, but only at the highest concentration (see Supplementary Fig. S3). Next, RAW264.7 cells were stimulated with the cathelicidins at various concentrations for 2 h and 24 h, after which release of CCL2 (MCP-1), CCL5 (RANTES), CXCL10 (IP-10), TNFα and IL-10 was determined.

**Figure 2:**
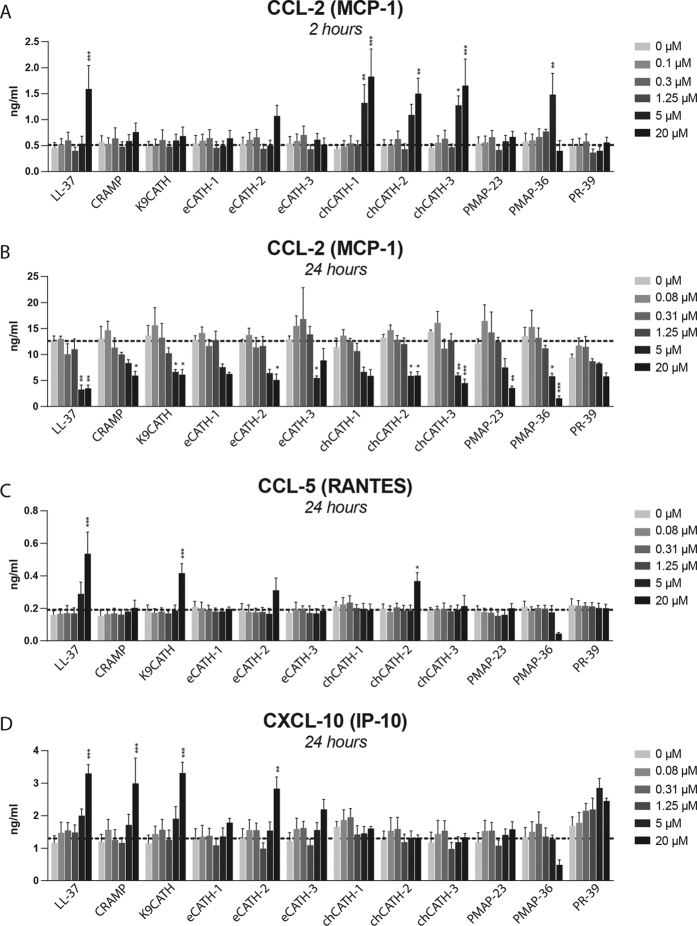
Cathelicidin induced chemokine and cytokine release by RAW264.7 cells. RAW264.7 cells were incubated for 2 h and 24 h with cathelicidins (0.08 μM, 0.31 μM, 1.25 μM, 5 μM, and 20 μM), after which the supernatants were harvested and tested for release of CCL2 at 2 h (**A**) and 24 h (**B**), CCL5 at 24 h (**C**) and CXCL10 at 24 h (**D**). Dotted line represents average cytokine release of control samples. Results are presented as average +/− SEM (N = 3). Statistical differences were determined by Two-way ANOVA with Bonferroni post-hoc test.

After 2 h stimulation CCL2 secretion was 3–4 fold enhanced by chCATH-1, chCATH-3 and PMAP-36 at 5 μM and by LL-37 and chCATH-1, -2 and -3 at 20 μM. None of the other peptides had a significant effect on CCL2 secretion after 2 h stimulation (Fig. 2A). In contrast, all cathelicidins inhibited CCL2 secretion after 24 h stimulation, although inhibition by eCATH-1, chCATH-1 and PR-39 was non-significant (Fig. 2B). CCL5 secretion after 24 h stimulation is 2–3 fold induced, however by a different set of cathelicidins than found for CCL2, e.g. LL-37, K9CATH and chCATH-2 (20 μM) (Fig. 2C). LL-37, CRAMP, K9CATH and eCATH-2 (20 μM) were able to induce CXCL10 secretion with a 2–3 fold increase after 24 h stimulation (Fig. 2D). Secretion of TNFα (2 h) and IL-10 (24 h) was also determined after cathelicidin stimulation. Some induction was observed at higher concentrations; however, the measured cytokine levels were very low. (see Supplementary Fig. S4A,B). Various cathelicidins significantly induce cytokine and chemokine secretion. This increase appears marginal in comparison to TNFα, CCL5 and IL-10 secretion after stimulation with viable or heat-killed *E. coli* (see Supplementary Fig. S4C), only the induction of CXCL10 secretion by cathelicidins was in the same range as after *E. coli* stimulation.

### Effects of cathelicidins on TLR-2, -4, and -9 activation

**Figure 3:**
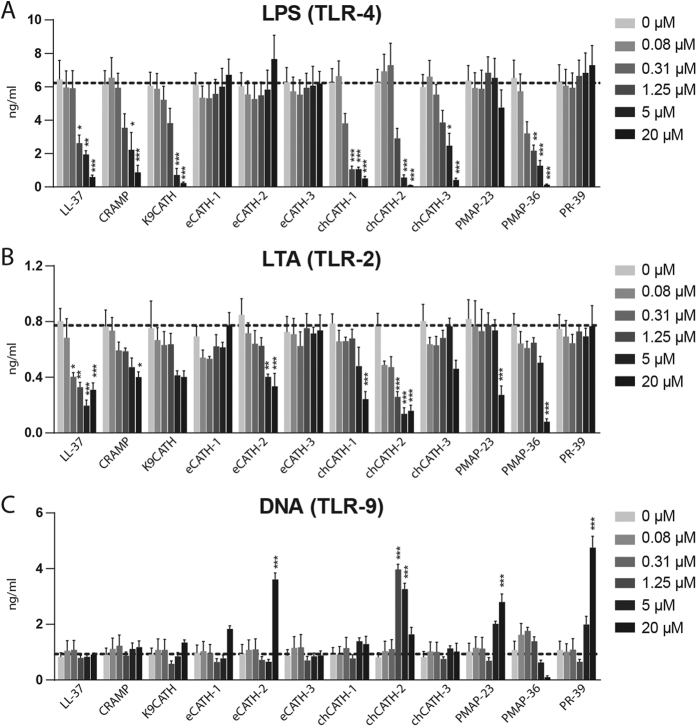
Effects of cathelicidins on TLR-2, -4, and -9 activation. LPS (100 ng/ml) (**A**), LTA (1 μg/ml) (**B**) or ODN-1826 (2.5 nM) (**C**) was mixed with different cathelicidins (0.08 μM, 0.31 μM, 1.25 μM, 5 μM, and 20 μM) before addition to the RAW264.7 cells. Supernatants were collected after 2 hours (**A,B**) or 24 hours (**C**) incubation and TNFα expression was measured. The dotted line represents the average cytokine release of control samples. Results are presented as average +/− SEM (N = 3). Statistical differences were determined by Two-way ANOVA with Bonferroni post-hoc test.

RAW264.7 cells were stimulated for 2 h with 100 ng/ml LPS from *E. coli* O111:B4 in the presence of different cathelicidins at various concentrations to study the capability of LPS neutralization (Fig. 3A). Seven out of 12 cathelicidins significantly neutralized LPS at a concentration of 1.25 μM or 5 μM and thereby inhibited LPS-induced activation. None of the equine cathelicidins nor PMAP-23 and PR-39 were capable to neutralize LPS. Next, RAW264.7 cells were stimulated with 1 μg/ml *S. aureus* LTA for 2 h in the presence of various cathelicidin concentrations, to test LTA neutralization (Fig. 3B). Also seven out of 12 cathelicidins were able to neutralize LTA; interestingly, while some cathelicidins, such as chCATH-2 and LL-37, potently inhibited both LPS- and LTA-induced activation, others only significantly inhibited either LPS, such as K9CATH and chCATH-3, or LTA, like eCATH-2.

Since, LL-37, CRAMP, chCATH-2, and PMAP-36 have been described to enhance DNA-induced activation^22^,^23^,^36^,^37^, the effect of all cathelicidins on DNA-induced TNFα release in RAW264.7 cells was analyzed (Fig. 3C). chCATH-2 was most potent to induce DNA activation after 24 h with an optimal concentration of 1.25 μM. eCATH-2, PMAP-23 and PR-39 (20 μM) were also able to enhance DNA-induced TLR-9 activation. All other peptides had no effect on the DNA-induced TLR-9 activation. To ensure that the increase of TNFα secretion was indeed enhancement of DNA-induced activation and not a result of direct induction by cathelicidins, TNFα release was determined after 24 h cathelicidin stimulation (see Supplementary Fig. S2D). None of the cathelicidins induced TNFα release after 24 h, (if anything, most cathelicidins caused a small reduction of TNFα secretion) indicating the increased activation is indeed enhancement of DNA-induced activation.

### Effects of cathelicidins on phagocytosis by RAW264.7 cells

**Figure 4:**
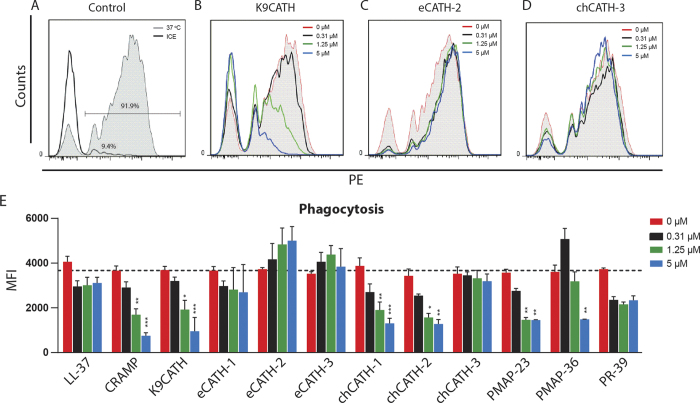
Effects of cathelicidins on phagocytosis by RAW264.7 cells. RAW264.7 cells were incubated with cathelicidins (0.31 μM, 1.25 μM, and 5 μM) and red fluorescent latex beads (10 beads to 1 cell) at 37 °C (energy dependent uptake) or on ice (non-specific adherence) for 30 minutes. Histograms show control (no peptide present) bead uptake at 37 °C (gray, filled) and 0 °C (black line) (**A**), or uptake in presence of different concentrations of indicated cathelicidins (**B–D**); 0 μM (red, filled), 0.31 μM (black line), 1.25 μM (green line) or 5 μM (blue line). Uptake was quantified by determining the MFI after correction for 0 °C control (**E**). Results are presented as average +/− SEM (N = 3). Statistical differences were determined by Two-way ANOVA with Bonferroni post-hoc test.

While the above-described functions of cathelicidins are well-known, relatively little is known about the influence of cathelicidins on phagocytosis^27^. Therefore, RAW264.7 cells were incubated with fluorescent beads to study the effect of cathelicidins on phagocytosis. The uptake was corrected for fluorescence measured after incubation on ice instead of 37 °C, which inhibits the active process of phagocytosis. After 30 minutes, 90% of the RAW264.7 cells took up latex beads (Fig. 4A). Histograms exemplify changes in bead phagocytosis with different concentrations of K9CATH (Fig. 4B), eCATH-2 (Fig. 4C) and chCATH-3 (Fig. 4D). Effect of all cathelicidins are depicted by the average mean fluorescence intensity (MFI) in Fig. 4E.

CRAMP, K9CATH, chCATH-1 and -2, and PMAP-23 reduced bead-uptake in a dose-dependent manner, whereas PMAP-36 only reduced phagocytosis at 5 μM. In contrast, eCATH-2 was the only peptide that increased the uptake by almost 50%, although not significantly. However, as shown in Fig. 4C, RAW264.7 cells are not be able to take up more beads per cell. LL-37, eCATH-1 and -3, chCATH-3, and PR-39 did not affect bead uptake at any of the used concentrations.

## Discussion

The current knowledge on functions of cathelicidins is mostly based on results from experiments with the human cathelicidin LL-37 and, to a lesser extent, murine CRAMP. In addition, even the most extensively described functions are often tested under different conditions, which makes it difficult to compare properties of cathelicidins. In this study, 12 cathelicidins were selected and compared in different assays to determine the conservation of the different functions between cathelicidins (results are summarized in Table 2). The cathelicidin selection included a number of well-known cathelicidins that have already been tested for various functions, such as LL-37, CRAMP, PR-39 and chCATH-2. In addition, several cathelicidins of which very little is known, such as the equine cathelicidins and K9CATH, were selected and compared to the better studied peptides. Furthermore, chCATH-1 and -3 were included to complement chCATH-2 and PMAP-23 and -36 were selected to represent the α-helical porcine cathelicidins. In fact, all selected cathelicidins are α-helical cathelicidins, except for PR-39, which is a proline-rich peptide forming a polyproline helix.

Antimicrobial activity of cathelicidins has been extensively tested over the years and has been demonstrated for all cathelicidins included in this study^35^,^38^,^39^,^40^,^41^,^42^,^43^,^44^,^45^,^46^. The antimicrobial capacities were tested under the same conditions and expressed as growth delay instead of the more classical MIC/MBC determination using broth dilution assays. The advantage of our methodology is that activity of less potent peptides that do not establish a real MBC value can still be determined and compared. As can be seen from Fig. 1A our C_max_ value does not correspond to MBC (since 20 μM LL-37 is below the MBC but reached our 10 hour delay criterium). However, by plating out wells without visible growth, it was shown that real MBC values were consistently close (0–4 fold difference) to the C_max_ indicating that the order of activity would be similar in classic broth dilution tests (results not shown). The only exception was PR-39 with a 16 fold higher MBC than C_max_ against *S. aureus* in DMEM. The fact that DMEM medium makes *S. aureus* susceptible to PR-39 is surprising since this peptide is thought to require active uptake by the SbmA transporter, only present in Gram – bacteria. The results obtained in this work, might indicate that the peptide possibly switches to a different mechanism of action with initially a bacteriostatic effect, but more thorough studies are required to elucidate the details of these initial observations.

The growth delay results indicate that most cathelicidins, except K9CATH and eCATH-3, have similar antimicrobial activity against *E. coli* (C_max_ of 5–20 μM or a growth delay of at least 9 hours at 20 μM). However, the antimicrobial potencies against MRSA strongly diverge. Interestingly, antimicrobial activity against *E. coli* is strongly reduced for all cathelicidins, except PMAP-36, if tested under more physiological conditions, i.e. DMEM + FCS, while activity against MRSA is enhanced for all cathelicidins under these conditions (Table 2). It has been shown in previous studies that salts or serum components of DMEM + FCS can lower cathelicidin antimicrobial activity for both Gram-positive and Gram-negative bacteria^29^,^30^,^41^,^47^. On the other hand, DMEM also contains carbonate, which can increase bacterial susceptibility to cathelicidin-mediated bacterial killing^48^. Although carbonate has been described to increase the susceptibility of *E. coli* towards cathelicidins, the presence of salts and serum might have a stronger inhibitory effect on the cathelicidins than carbonate on *E. coli*. For example, Ca^2+^ is important for the structural integrity of the outer membrane of Gram-negatives^49^. In contrast, the increased susceptibility by MRSA due to the carbonate probably has more influence than the inhibitory effects of salt and serum on the cathelicidins. In addition, additive or synergistic effects between serum components and cathelicidins might be another cause for the more efficient killing of *S. aureus* in DMEM + FCS^30^,^50^. These results suggest that, while most cathelicidins have antimicrobial activity, the efficacy is strongly dependent on the pathogen and the physiological conditions.

Murine RAW264.7 cells were used in all mammalian cell related assays. RAW264.7 cells have been used extensively to identify and describe a wide variety of cathelicidin functions, such as cytokine and chemokine induction, LPS-neutralization, LTA-neutralization, and DNA-enhancement, which were shown for multiple cathelicidins from various species, including human, mouse, pig, cow and chicken^18^,^23^,^31^,^32^,^33^,^37^,^51^,^52^,^53^,^54^,^55^,^56^. Although use of a murine cell line can obscure possible species-specific effects or cell-specific effects, the results can be used as a basis for further studies on cathelicidin-mediated effects in this cell line or as a comparison with primary cells from different species.

Similar to the extensive research on antimicrobial activity, LPS neutralization has been shown in multiple studies for at least 13 different cathelicidins from 9 different species^18^,^43^,^45^,^57^,^58^,^59^,^60^,^61^,^62^,^63^,^64^,^65^,^66^. Therefore, it is thought to be one of the main cathelicidin functions. However, to our knowledge, nothing is known yet about the LPS neutralizing activity of canine, equine and porcine cathelicidins. Our results showed that 7 out of 12 cathelicidins inhibited LPS-induced macrophage activation, including K9CATH and PMAP-36, but none of the equine cathelicidins. Also 7 cathelicidins were found to neutralize LTA; however, there appears to be no correlation between LPS neutralization and LTA neutralization. For instance, LL-37 and CATH-2 potently exert both functions, while eCATH-2 only inhibited LTA-induced activation and K9CATH and chCATH-3 only showed potent inhibition of LPS-induced activation. In addition, neutralization of LPS and LTA did not appear to correlate with the antimicrobial activity against *E. coli* and MRSA, respectively (Table 2). These results showed that, while antimicrobial activity and LPS neutralization are commonly regarded as intrinsic properties of cathelicidins, these functions may differ between the various cathelicidins and species.

The induction of chemokine release by cathelicidins was first detected in RAW264.7 cells and was later also observed in THP-1 cells, primary monocytes and bronchial epithelial cells^18^,^67^,^68^,^69^. Our results indicate that several cathelicidins induced a 2–4 fold increase in chemokine expression by RAW264.7 cells at 20 μM; however, only LL-37 was able to increase the expression of all cytokines and chemokines tested (Table 2). The levels of chemokine and cytokine secretion induced by cathelicidins was generally low, especially compared to stimuli such as live or heat-killed *E. coli*. This appears to be in line with other studies, where cathelicidin-mediated induction of chemokine release in RAW264.7 or THP-1 cells also appears to be low compared to other stimuli, such as LPS^55^,^68^. In addition to the induction of chemokine release, it has been previously shown that cathelicidins can have a direct chemotactic effect^19^,^70^,^71^. The induction of low chemokine levels could be another explanation for the stimulation of chemotaxis by CRAMP and LL-37^72^,^73^. However, LL-37 has been shown to increase neutrophil influx in a murine lung model during inflammation, but without alteration of cytokine or chemokine expression^74^. Further research will be needed to understand to what extent direct chemotaxis and chemokine induction play a role in leukocyte recruitment during both steady state situations and in the context of an infection.

So far, antimicrobial activity and LPS neutralization, but not cytokine and chemokine induction, appear to be major cathelicidin functions, although not conserved for all cathelicidins. Enhanced DNA-induced TLR9 activation has been described in literature for LL-37, CRAMP, chCATH-2 and PMAP-36. Our results show that enhancement of DNA-induced macrophage activation is not a conserved function of cathelicidins, but only found for eCATH-2, chCATH-2, PMAP-23, and PR39, with chCATH-2 as most potent one (Table 2). chCATH-2 has previously been described to enhance macrophage activation due to increased DNA uptake^23^. LL-37, on the other hand, was shown to form a complex with DNA which enhances binding efficiency and increased IFNα production in pDCs^75^ and monocytes^76^. In B-cells, LL-37 enhances the uptake of DNA and promotes IL-6 production^77^. Endogenous CRAMP has been shown to increase TLR9 activation and TNFα release in macrophages; however, exogenous treatment with CRAMP had no effect on TLR-9 activation^37^. Together with the results presented in this study, it appears that the presence of exogenous cathelicidins can enhance DNA-induced stimulation; however, in a species- and cell type-specific manner.

Because relatively little is known about the influence of cathelicidins on phagocytosis, an initial analysis on phagocytosis was performed. Six out of 12 cathelicidins reduced latex bead internalization with eCATH-2 as only the only cathelicidin that induced uptake (Table 2). However, since uptake of extracellular components is a complex process, it is not possible to draw conclusions about functions *in vivo* based on these initial observations only^78^. Nevertheless, a detailed analysis with more specific inhibitors for phagocytosis, such as cytochalasin D, and live bacteria with or without opsonization, could lead to a more detailed understanding of the role of cathelicidins in the regulation of phagocytosis.

Finally, elucidation of cathelicidin functions is also important for the development of cathelicidin-based antibiotics. Due to the emergence of more multidrug resistant bacteria, new molecules with broad-spectrum antimicrobial activity could be useful to combat infections by antibiotic resistant bacteria, such as MRSA^79^. Especially chCATH-2 appears to be an interesting candidate with strong antimicrobial activity against both *E. coli* and MRSA under physiological conditions and, importantly, has been shown to induce only limited resistance in bacteria^80^. The dual activity of chCATH-2, i.e. antimicrobial activity and neutralization of LPS and LTA, can potentially provide protection against the infection as well as limit excessive inflammation. The latter is most important since sepsis is a major and life threatening problem in patients suffering from bacterial infections^81^.

In conclusion, this study provides a systematic comparison of 12 cathelicidins from 6 species, showing that physiological conditions can both positively and negatively affect antimicrobial activity and that the antimicrobial activity and LPS/LTA neutralization appear to be the most prevalent cathelicidin functions. However, this study also underlines the importance of not generalizing cathelicidin functions and that caution should be taken in the extrapolation of different functions, for instance the extrapolation from murine CRAMP KO-models to the human situation or other animal models.

## Additional Information

**How to cite this article**: Coorens, M. *et al*. Interspecies cathelicidin comparison reveals divergence in antimicrobial activity, TLR modulation, chemokine induction and regulation of phagocytosis. *Sci. Rep.* **7**, 40874; doi: 10.1038/srep40874 (2017).

**Publisher's note:** Springer Nature remains neutral with regard to jurisdictional claims in published maps and institutional affiliations.

## Supplementary Material

Supplementary Information

## Figures and Tables

**Table 1:** Cathelicidin sequences and antimicrobial activity.

Peptide	Sequence	Length	Charge	*E. coli*				MRSA			
				MHB		DMEM		MHB		DMEM	
				C_max_	Max	C_max_	Max	C_max_	Max	C_max_	Max
LL-37	LLGDFFRKSKEKIGKEFKRIVQRIKDFLRNLVPRTES	37	+6	**10** μ**M**		—	4.5 hr	—	0.5 hr		9.5 hr
CRAMP	GLLRKGGEKIGEKLKKIGQKIKNFFQKLVPQPEQ	34	+6	—	9.0 hr	—	2.5 hr	—	0.5 hr	—	6.5 hr
K9CATH	RLKELITTGGQKIGEKIRRIGQRIKDFFKNLQPREEKS	38	+6	—	3.0 hr	—	1.5 hr	—	0.5 hr	—	4.5 hr
eCATH-1	KRFGRLAKSFLRMRILLPRRKILLAS	26	+9	**20** μ**M**		—	3.5 hr	—	7.5 hr	**20** μ**M**	
eCATH-2	KRRHWFPLSFQEFLEQLRRFRDQLPFP	27	+4	—	9.0 hr	—	2.0 hr	—	1.5 hr	—	3.5 hr
eCATH-3	KRFHSVGSLIQRHQQMIRDKSEATRHGIRIITRPKLLLAS	40	+10	—	0.0 hr	—	1.5 hr	—	0.0 hr	—	1.5 hr
chCATH-1	RVKRVWPLVIRTVIAGYNLYRAIKKK	26	+8	**10** μ**M**		—	9.5 hr	**2**.**5** μ**M**		**1**.**25** μ**M**	
chCATH-2	RFGRFLRKIRRFRPKVTITIQGSARF-NH_2_	26	+9	**5** μ**M**		**10** μ**M**		**2**.**5** μ**M**		**0**.**3** μ**M**	
chCATH-3	RVKRFWPLVPVAINTVAAGINLYKAIRRK	29	+7	**20** μ**M**		—	5.5 hr	**5** μ**M**		**0**.**3** μ**M**	
PMAP-23	RIIDLLWRVRRPQKPKFVTVWVR	23	+6	—	9.5 hr	—	3.5 hr	**20** μ**M**		**10** μ**M**	
PMAP-36	Ac-GRFRRLRKKTRKRLKKIGKVLKWIPPIVGSIPLGCG	36	+13	**5** μ**M**		**2**.**5** μ**M**		**10** μ**M**		**0**.**3** μ**M**	
PR-39	RRRPRPPYLPRPRPPPFFPPRLPPRIPPGFPPRFPPRFP	39	+10	**20** μ**M**			7.0 hr	—	0.5 hr	**10** μ**M**	

**C**_**max**_: cathelicidin concentration (μM) resulting in a delay in bacterial growth with minimal 10 hours.

**Max**: if no **C**_**max**_ was reached, hours growth delay was depicted with 20 μM cathelicidin.

**Table 2:** Summary of cathelicidin functions.

Peptide	*E. coli*		*S. aureus*		TLR activation			Chemokines			Phagocytosis
	MHB	DMEM	MHB	DMEM	LPS	LTA	DNA	CCL2 (2 h)	CCL5 (24 h)	CXCL10 (24 h)	
LL-37	+++	+	−	++	↓↓	↓↓	−	↑	↑	↑	−
CRAMP	++	−	−	+	↓↓	↓	−	−	−	↑	↓↓
K9CATH	−	−	−	+	↓↓	−	−	−	↑	↑	↓↓
eCATH-1	+++	−	+	+++	−	−	−	−	−	−	−
eCATH-2	++	−	−	−	−	↓↓	↑	−	−	↑	−
eCATH-3	−	−	−	−	−	−	−	−	−	−	−
chCATH-1	+++	++	+++	+++	↓↓	↓	−	↑↑	−	−	↓↓
chCATH-2	+++	+++	+++	+++	↓↓	↓↓	↑↑	↑	↑	−	↓↓
chCATH-3	+++	+	+++	+++	↓↓		−	↑↑	−	−	−
PMAP-23	++	−	+++	+++	−	↓	↑	−	−	−	↓↓
PMAP-36	+++	+++	+++	+++	↓↓	−	−	↑	−	−	↓↓
PR-39	+++	+	−	+++	−	−	↑	−	−	−	−

+++ = C_max_ at ≤20 μM, ++ = >8 hours inhibition at 20 μM, + = >4 hours inhibition at 20 μM.

↑↑ = significant increase ≤5 μM, ↑ = significant increase at 20 μM.

↓↓ = significant decrease ≤5 μM, ↓ = significant decrease at 20 μM.
