# Supplementary material for: Interspecies cathelicidin comparison reveals divergence in antimicrobial activity, TLR modulation, chemokine induction and regulation of phagocytosis
Source: Sci Rep. 2017 Jan 19;7:40874. doi: 10.1038/srep40874 (PMC5244392; doi:10.1038/srep40874)
Supplement: Supplementary Information [file srep40874-s1.pdf]

Supplementary files

Interspecies cathelicidin comparison reveals divergence in antimicrobial activity, TLR modulation, chemokine induction and regulation of phagocytosis.

Maarten Coorens[#], Maaike R. Scheenstra[#], Edwin J. A. Veldhuizen, Henk P. Haagsman[*]

Department of Infectious Diseases and Immunology, Division of Molecular Host Defence, Faculty of Veterinary Medicine, Utrecht University, Yalelaan 1, 3584 CL Utrecht, The Netherlands

Footnotes

[#]These authors contributed equally to this paper

[*]To whom correspondence may be addressed. E-mail: H.P.Haagsman@uu.nl. Telephone: +31302535354.

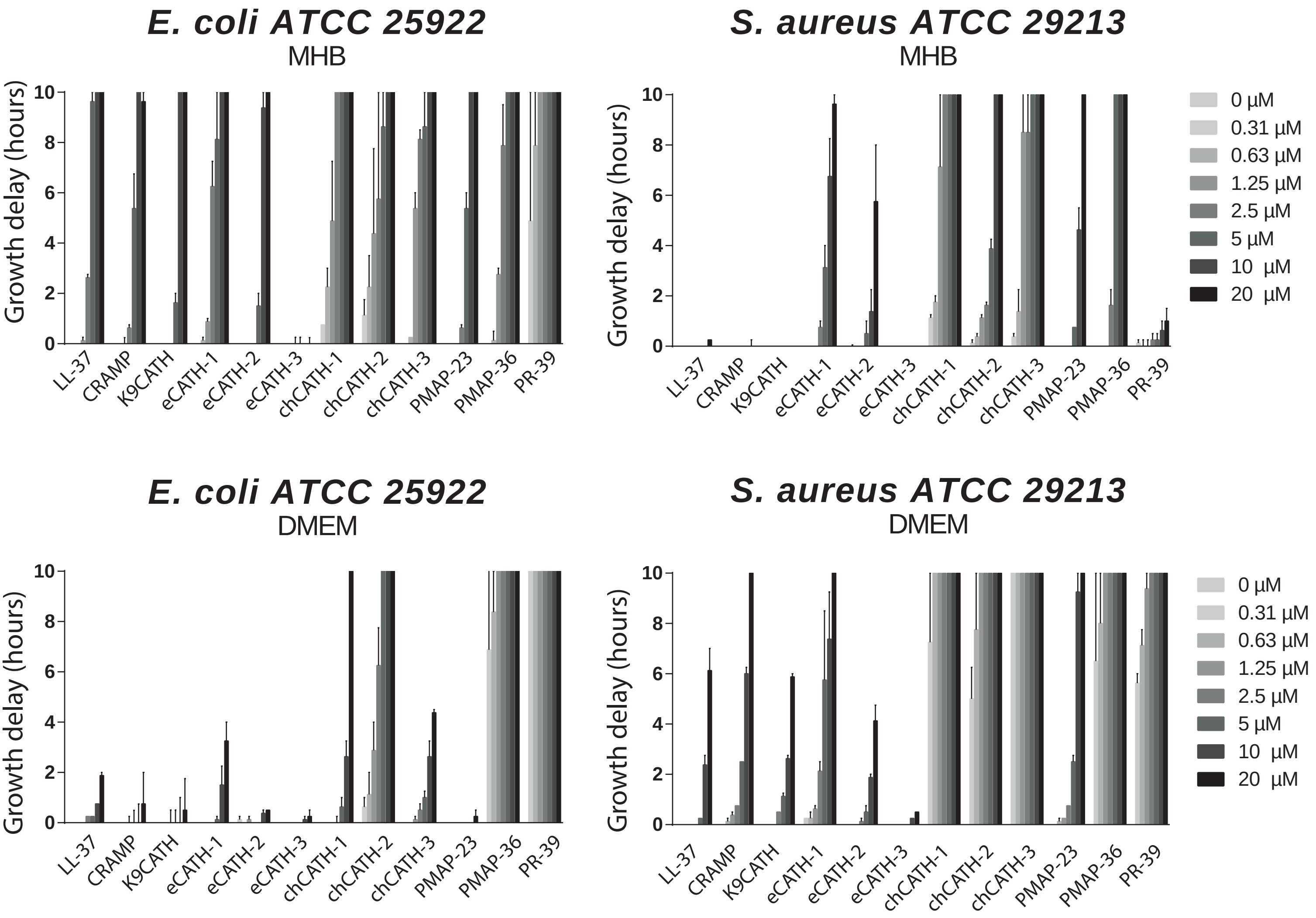

Supplementary Figure 1: Antibacterial activity of cathelicidins

E. coli ATCC 25922 and S. aureus ATCC 29213 (1x106 CFU/ml) were grown in MHB or DMEM+FCS for 16 hours under constant shaking (200 RPM) in the presence of different concentration of cathelicidins (0.31 µM, 0.63 µM, 1.25 µM, 2.5 µM, 5 µM, 10 µM, and 20 µM). The OD was measured every 15 minutes. Growth delay was defined as the time needed for peptide-treated bacteria to grow above an OD of 0.6 compared to the control bacteria (no peptide added). Results are presented as average +/- SEM (N=2).

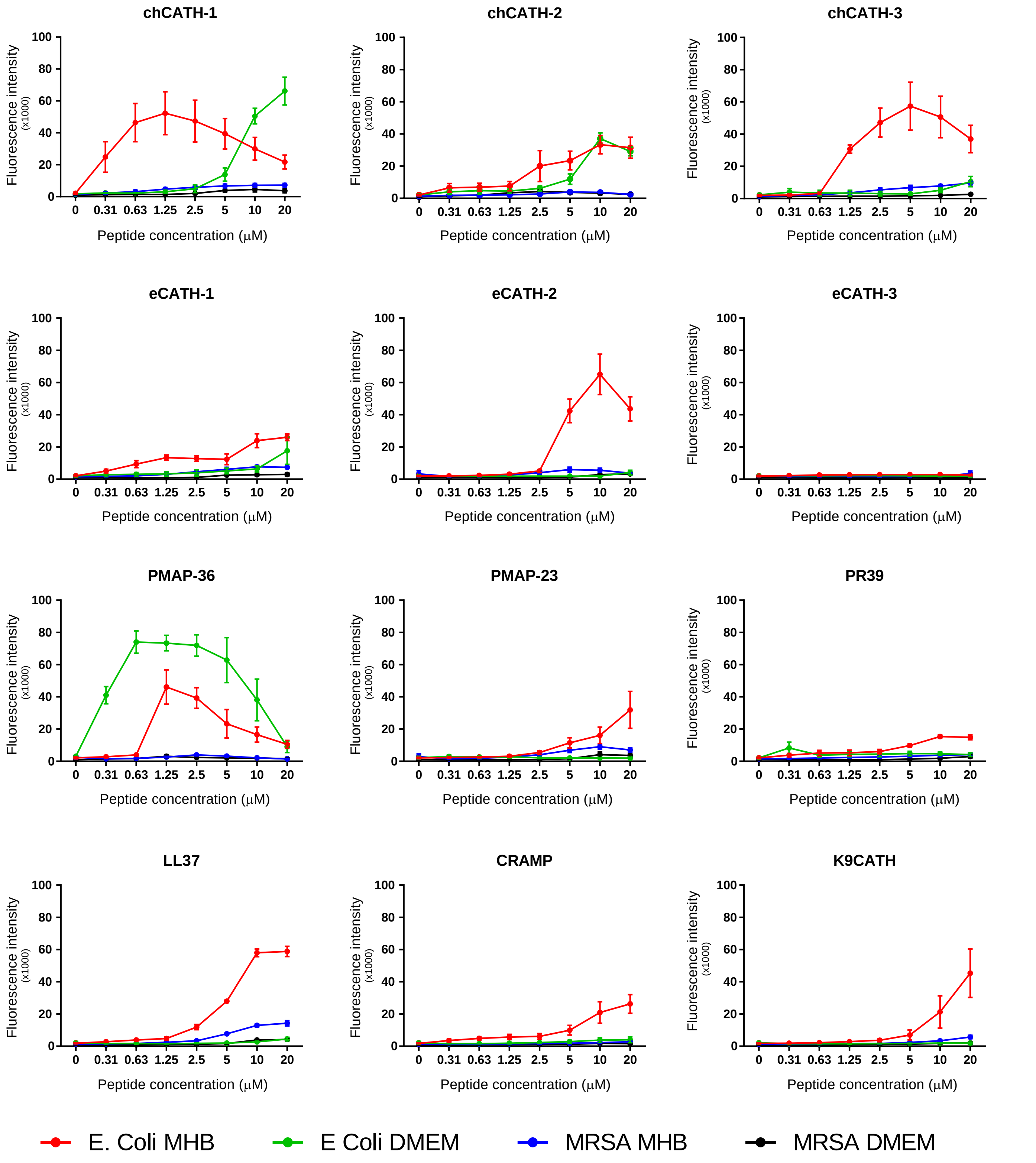

Supplementary Figure 2: Bacterial membrane leakage

E. Coli O78 and MRSA (1x10^6 CFU/ml) were grown in MHB or DMEM+FCS for 30 minutes at 37°C in the presence of different concentrations of cathelicidins (0.31 µM, 0.63 µM, 1.25 µM, 2.5 µM, 5 µM, 10 µM, and 20 µM), after which the cells were stained with sytox green to measure membrane leakage. E. Coli in MHB, dark green line; E. Coli in DMEM, light green line; MRSA in MHB, dark blue line; MRSA in DMEM, light blue line. Results are presented as average +/- SEM (N = 3).

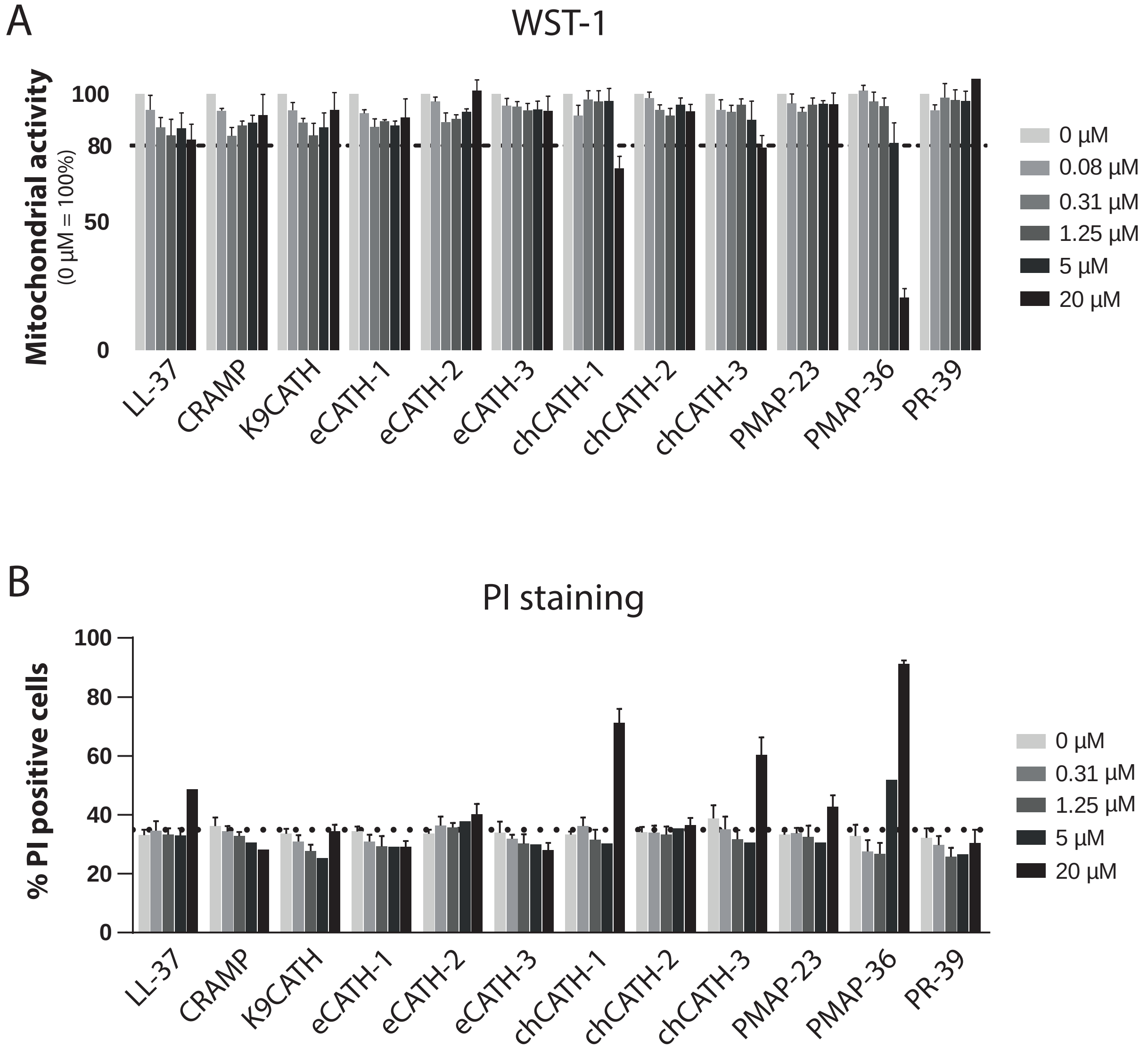

Supplementary Figure 3: Cytotoxicity of cathelicidins on RAW264.7 cells

(A) RAW264.7 cells were incubated for 24 hours with different cathelicidins (0.08 µM, 0.31 µM, 1.25 µM, 5 µM, and 20 µM), after which they were incubated with WST-1 reagent for 20 minutes to measure the mitochondrial activity. No peptide control was set to 100% mitochondrial activity. Dotted line represents 80% mitochondrial activity. Results are presented as average +/- SEM (N = 4).

(B) RAW264.7 cells were incubated with different cathelicidins (0.31 µM, 1.25 µM, 5 µM, and 20 µM). Cells were stained for PI to detect dead cells. Dotted line represents average (34%) PI positive cells in the 0 µM control. Results are presented as average +/- SEM (N = 3).

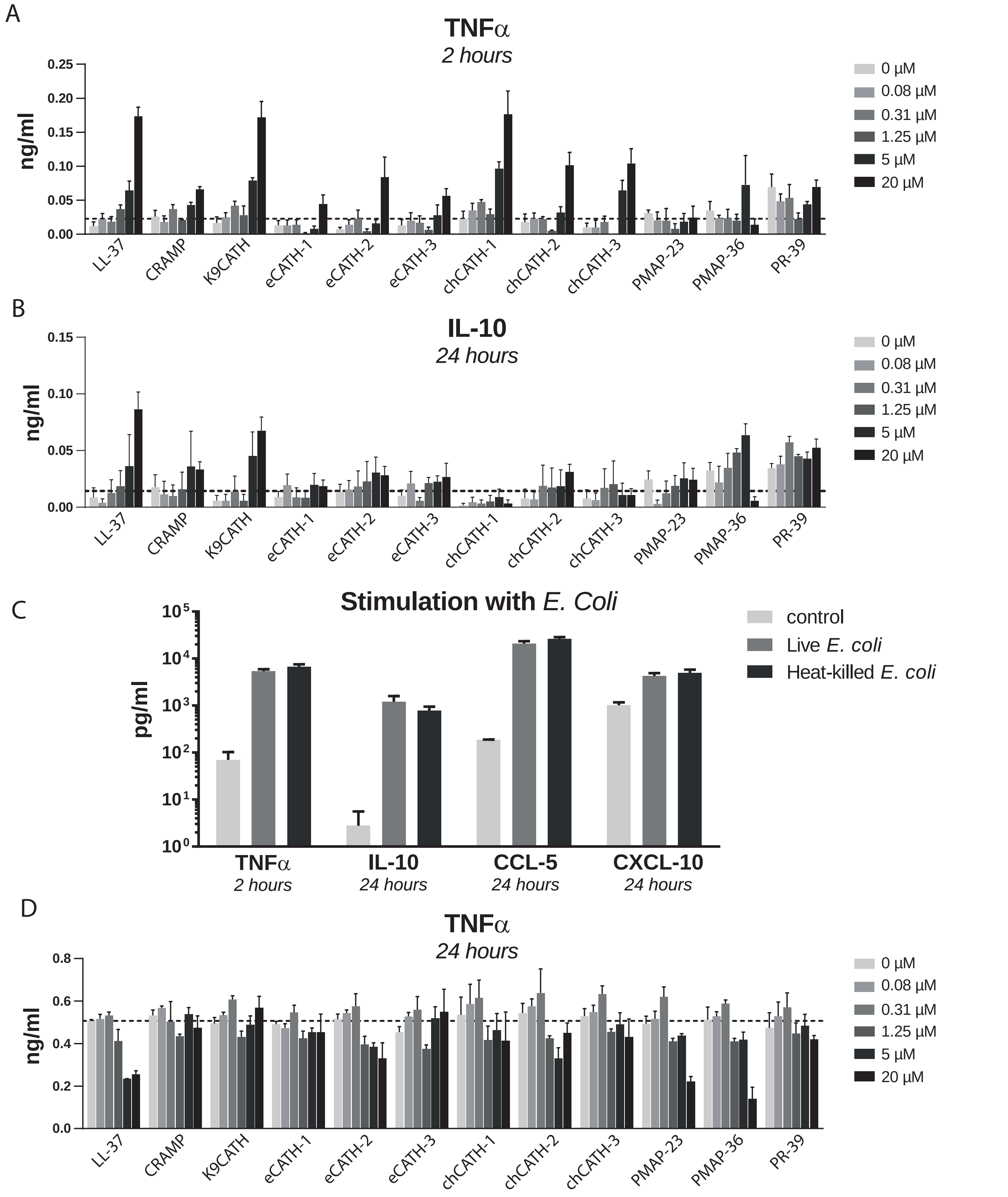

Supplementary Figure 4: Cathelicidin induced chemokine and cytokine release by RAW264.7 cells
RAW264.7 cells were incubated with cathelicidins (0.08 μM, 0.31 μM, 1.25 μM, 5 μM, and 20 μM), after which the supernatants were tested for TNFα after 2 h (A) and IL-10 after 24 h (B). Stimulation of RAW264.7 cells with 10^6 CFU/ml live or heat-killed (70 °C, 30 minutes) E. coli O78 for 2 h, after which cells were washed three times and incubated for another 22 h with culture media containing 250 μg/ml gentamicin. TNFα release was determined after 2 h and IL-10, CCL5 and CXCL10 release was determined after 24 h (C). RAW264.7 cells were incubated for 24 h with cathelicidins (0.08 μM, 0.31 μM, 1.25 μM, 5 μM, and 20 μM), after which the supernatants were tested for TNFα concentrations (D). Results are presented as average +/- SEM (N = 3).
